# Characterization of *Cellulose synthase-like D* (*CSLD*) family revealed the involvement of *PtrCslD5* in root hair formation in *Populus trichocarpa*

**DOI:** 10.1038/s41598-018-36529-3

**Published:** 2019-02-05

**Authors:** Xiaopeng Peng, Hongying Pang, Manzar Abbas, Xiaojing Yan, Xinren Dai, Yun Li, Quanzi Li

**Affiliations:** 1 State Key Laboratory of Tree Genetics and Breeding, Chinese Academy of Forestry, Beijing, 100091 China; 2 Research Institute of Forestry, Chinese Academy of Forestry, 100091 Beijing, China; 3 National Engineering Laboratory for Tree Breeding, College of Biological Sciences and Technology, Beijing Forestry University, Beijing, 100083 China

## Abstract

*Cellulose synthase-like D* (*CSLD*) family was characterized for their expression and functions in *Populus trichocarpa*. Ten members, *PtrCslD1-10*, were identified in the *P. trichocarpa* genome, and they belong to 4 clades by phylogenetic tree analysis. qRT-PCR and promoter:GUS assays in Arabidopsis and *P*. *trichocarpa* displayed divergent expression patterns of these 10 *PtrCSLD* genes in root hairs, root tips, leaves, vascular tissues, xylem and flowers. Among *PtrCslD2*, *PtrCslD4*, *PtrCslD5*, *PtrCslD6*, and *PtrCslD8* that all exhibited expression in root hairs, only *PtrCslD5* could restore the root hairless phenotype of the *atcsld3* mutant, demonstrating that *PtrCslD5* is the functional ortholog of *AtCslD3* for root hair formation. Our results suggest more possible functions for other *PtrCslD* genes in poplar.

## Introduction

Root hairs are integral for anchorage, enlarging surface area for absorption of water and nutrients, symbiosis interface between plants and soil biome, and expanding exploited soil area to avoid soil erosion^1,2^. Arabidopsis root hairs have been serving as a model to study cellular morphogenesis, such as plant cell growth and tip growth^3^. Trichoblast cell’s basal ends of root epidermis specialized to give rise to bulges, which serve as primordia and elongate into thin tubular structures called root hairs^4,5^. Trichoblasts progressively divide and expand specialized elongating cells at the tip-growing pole^6^. Differentiating morphological stages of root hairs include specification, initiation, elongation and cessation^1,3^. A number of genes such as *AthA*, *AthB*, *CPC, ROP, RSW1*, *RHD*, *COW*, *TIP*, *CEN*, *SCN* and *BST* play key roles in root hair tip growth^7–10^. For examples, *CPC* promotes trichoblast cell differentiation, *AtROP2* and *AtROP4* are key factors in bulge initiation^11,12^, *COW1* (*CAN OF WORMS*), *TIP1*, *CEN1* (*CENTEPEDE*), *CEN2*, *CEN3* and *BST1* (*BRISTLED*) control one root hair per trichoblast^7,9,13^. Rapid polarized exocytosis by cell division at the root hair tip supports cell wall maintenance by deposition of cellulose, and loss in any cellulose component would lead to rupture and ectopic root hair formation^1,14^. To elucidate gene functions in root hair formation, genetic analysis are required^5^.

The cellulose synthase like (*CSL*) gene superfamily is composed of 30 genes in Arabidopsis, which encode glycosyltransferases for biosynthesis of polysaccharides and have tissue-specific expression patterns^15–18^. Based on the sequences, the *CSL* gene family is divided into *CSLA* to *CSLG* groups^19^. *CSLA, CSLC* and *CSLF* are involved in mannan, xyloglucan, and (1 → 3; 1 → 4)-β-D-glucan biosynthesis, respectively^15,20–25^. The *CSLD* family member shares high amino acid similarity with the *CESA* family and is involved in root tip formation^22,25^. Abnormal flowers, pollen tubes and pollen grains were observed in *atcsld1*, *atcsld4* and *nacsld3* mutants^26–28^, while *atcsld2*, *atcsld3*, *atcsld5*, *oscsld1* and *oscsld4* mutant seedlings were root hairless^16,29–31^. *PtrCslD2*, an ortholog of *AtCslD3*, showed its expression level in xylem also^32^.

Yin *et al*. developed *atcsld2*/*csld3*, *atcsld2*/*csld5*, *atcsld3*/*csld5* and *atcsld2*/*csld3*/*csld5* double and triple *Arabidopsis* knockout mutants. All mutants were dwarf and displayed severe necrosis, indicating the collaborative effects among *AtCslD2*, *AtCslDd3* and *AtCslD5*^33^. The expression of *AtCslD2* is *AtCslD3-*dependent, and the defects in the *atcsld3* mutant were partially compensated for by *AtCslD2* overexpression^14^. Two other *CSLD* genes, *PdCslD5* and *PdCslD6*, were complementation remedies for defects and abnormalities of *atcsld3* mutants, which proved that the aforementioned genes are functional orthologs of the *AtCslD3*^34^.

*AtCslD1* and *AtCslD4* are responsible for cellulose deposition in cell walls to avoid ectopic pollen tubes and pollen grains^35^. *AtCslD3* is crucial for the tensile strength of root hair tip cells by deposition of cellulose, and *atcsld3* mutants were unable to maintain homeostasis, and terminated bulge elongation at early stage was observed^14,36^. Stunted root and shoot growth, a decreased concentration of homoglacturonan and xylans, and an elevated concentration of the cellulose synthase inhibitor isoxaben were observed in the *atcsld5* mutants^15^. The rice *oscsld1* mutant had normal root hair initiation, but displayed stunted root hair growth, swelling and kinking, showing that *OsCslD1* is a functional ortholog of *AtCslD3*/*KOJAK*/*RHD7* and functioning in root hair elongation^30^. Retarded growth and arrested cell division due to lack of cellulose deposition in culm and root tips of rice *nd1* mutants (*OsCslD4*) was observed^37^.

Root and root hair growth have already been explored in maize, rice, cotton and Arabidopsis^14,37–39^. In this study, we identified 10 *CSLD* genes (*PtrCslD1*-*10*) in *P. trichocarpa* and investigated their possible functions. We studied their expression pattern by qRT-PCR and promoter::GUS staining, and their involvement in root hair formation was investigated by complementation in the Arabidopsis *atcsld3* mutant. We demonstrate the functions of *PtrCslD5* in root hair formation and provide preliminary evidence of the involvement of *CSLD* members in xylem formation.

## Results

### Characterization of the *CSLD* family in *Populus trichocarpa*

We used Arabidopsis *CSLD* gene sequences to BLASTN (E-value ≤ 1.0) *P. trichocarpa* genome and obtained 10 homologous gene models. We named these genes *PtrCslD1* (Potri.002G200300), *PtrCslD2* (Potri.014G125100), *PtrCslD3* (Potri.003G097100), *PtrCslD4* (Potri.001G136200), *PtrCslD5* (Potri.019G046700), *PtrCslD6* (Potri.013G082200), *PtrCslD7* (Potri.004G208800), *PtrCslD8* (Potri.009G170000), *PtrCslD9* (Potri.003G177800), and *PtrCslD10* (Potri.001G050200). To understand dynamic topological evolution, a neighbor joining phylogenetic tree was constructed by MEGA 7.0^40–42^ using *CSLD* genes, including the above *P. trichocarpa CSLD* genes and the *CSLD* gene in Arabidopsis^7,14,15,31,33,35,43,44^, rice, cotton and maize^30,34,37^. Phylogenetic tree analysis and amino acid sequence comparison among these 10 genes belong to four clades (I–IV). Among these 10 *PtrCslD* genes, two genes in the same clade had ~89–91% sequence identity, indicating that they belong to gene pairs, probably formed by chromosome duplication^45,46^.

**Figure 1:**
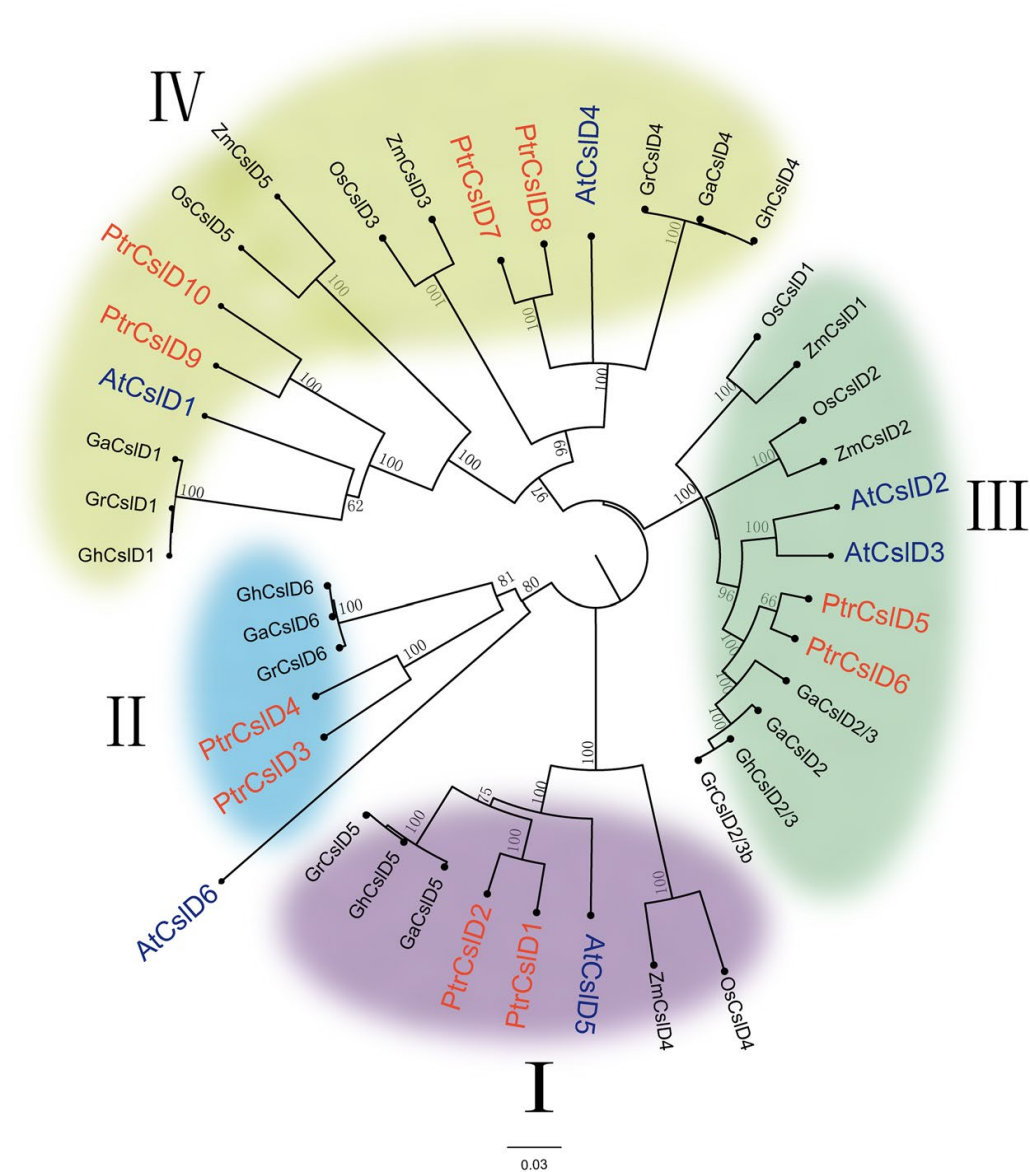
Phylogenetic tree of *CSLD* genes. A neighbor-joining (NJ) tree was constructed by MEGA 7.0 using 42 *CSLD* genes, including 10 genes from *Populus trichocarpa*, 6 genes from Arabidopsis, 5 genes from rice, 5 genes from maize, and 16 genes from cotton. The tree shows 4 distinct clades.

On phyletic lineage, gene pair *PtrCslD1* and *PtrCslD2* shared the same clade I with *ZmCslD4*, *OsCslD4*, *AtCslD5*, *GaCslD5*, *GhCslD5*, and *GrCslD5* (Fig. 1). In this clade, functions of *OsCslD4* and *AtCslD5* were studied, and both mutants displayed defective root hairs^15,37^. In clade II, *PtrCslD3* and *PtrCslD4* shared the same lineage with *AtCslD6*, *GrCslD6*, *GaCslD6*, *GhCslD6*; none of those were identified for their functions*. PtrCslD5* and *PtrCslD6* belong to gene pair, sharing high similarity with *AtCslD2*, *AtCslD3*, *OsCslD1*, *OsCslD2*, *GrCslD2*, *GaCslD2*, *GhCslD2*, *NaCslD3*, *ZmCslD1* and *ZmCslD2* in clade III. Among these 9 genes, *AtCslD2*, *AtCslD3*, *OsCslD1* are required for root hair morphogenesis, and *ZmCslD1* is essential for cell division of rapidly growing tissues^14,29,30,43,47^. Gene pair *PtrCslD7* and *PtrCslD8* and gene pair *PtrCslD9* and *PtrCslD10* belong to clade IV. *PtrCslD7* and *PtrCslD8* are closely related to *AtCslD4*, and *PtrCslD9* and *PtrCslD10* are closely related to *AtCslD1*. Mutation of both *AtCslD1* and *AtCslD4* caused abnormal flowers, pollen tubes, and pollen grains^35^. Based on the phylogenetic tree analysis, *PtrCslD1*, *PtrCslD2*, *PtrCslD5* and *PtrCslD6* may function in root hair formation, and *PtrCslD7*, *PtrCslD8*, *PtrCslD9* and *PtrCslD10* may participate in flower and pollen tube development.

### Expression patterns of *CSLD* members in *P. trichocarpa* plants

Understanding the gene expression pattern can give some clue as to their possible functions. We used quantitatively RT-PCR to examine the expression patterns of these *CSLD* genes in *P. trichocapra*. Their absolute transcript abundance in young roots, mature roots, young stem, leaves, xylem, phloem, and apex were determined. Considering the high nucleotide similarity between gene pairs, we designed specific primers to distinguish the gene pairs.

**Figure 2:**
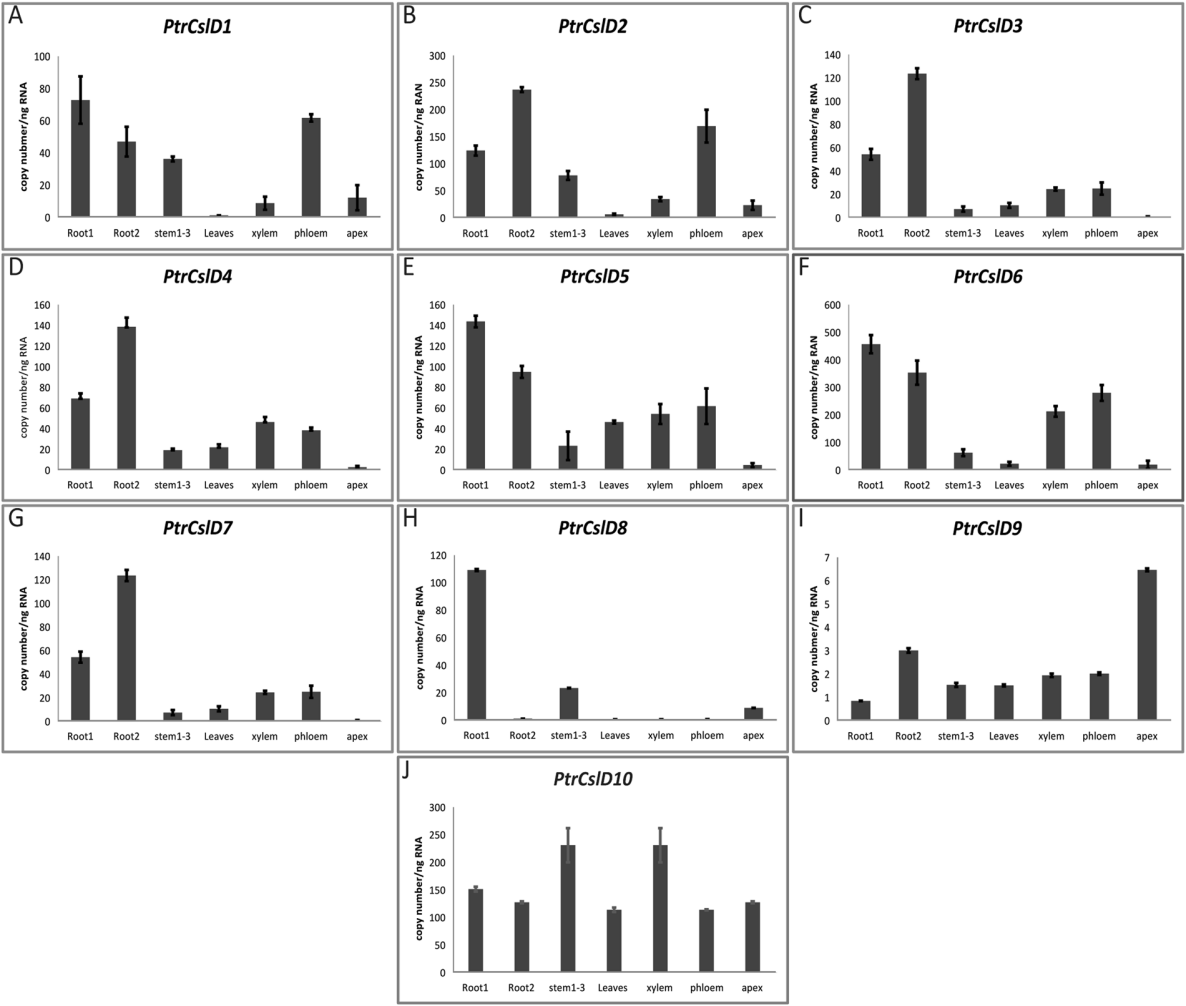
Expression analyses of *PtrCSLD* genes in *P. trichocarpa*. Absolute transcript abundance of *PtrCslD1* (**A**), *PtrCslD2* (**B**), *PtrCslD3* (**C**), *PtrCslD4* (**D**), *PtrCslD5* (**E**), *PtrCslD6* (**F**), *PtrCslD7* (**G**), *PtrCslD8* (**H**), *PtCslD9* (**I**), and *PtrCslD10* (**J**), were examined in young roots (root 1), mature roots (root 2), stems of internodes 1–3 (stem 1–3), leaves, xylem, phloem, and shoot apex. The plasmid containing the gene was used as a standard for establishing a quantitative correlation between the copy number of the target gene transcript molecules and the C_T_ values. Error bars represent standard errors of triplicate assay.

*PtrCslD1* and *PtrCslD2* displayed similar expression patterns, with high expression levels in young roots, mature roots, young stems, and phloem, while with relatively low levels in xylem and apex, and the lowest level in leaves. However, *PtrCslD1* and *PtrCslD2* displayed inverse expression levels between young and mature roots (Fig. 2A,B). A comparatively higher transcript abundance of *PtCslD3*, 4, 5 and 6 was detected in roots than in young stems, xylem and phloem, and apex had low transcript abundance (Fig. 2C–F). A high expression level in roots was observed for *PtrCslD7* and *PtrCslD8. PtrCslD7* was highly expressed in both young and mature roots, while *PtCslD8* was detected only for its expression in young roots (Fig. 2G,H). *PtrCslD9* and *PtrCslD10* were expressed in all tissues, but absolute transcript abundance of *PtrCslD10* was very low in these tissues (Fig. 2I,J). Although similar expression patterns between each gene pair were generally observed, in some tissues the gene pair exhibited variable expression levels. For example, high transcript abundance was detected in mature roots for *PtrCslD7* but not for *PtrCslD8*.

### Expression analysis by promoter::GUS staining

**Figure 3:**
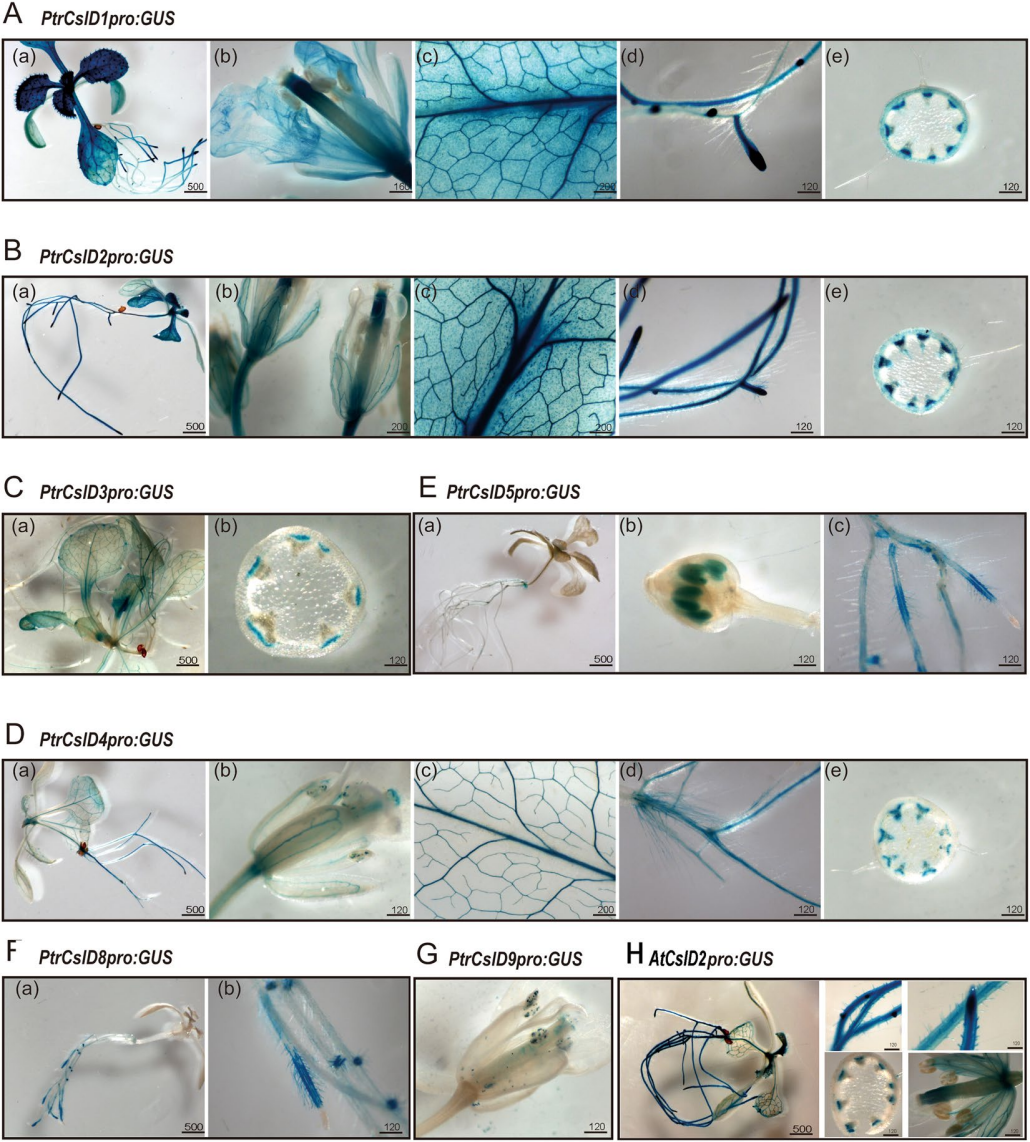
GUS staining of promoter:GUS in Arabidopsis. *PtrCslD1* promoter:GUS (**A**). *PtrCslD2* promoter:GUS (**B**). *PtrCslD3* promoter:GUS (**C**). *PtrCslD4* promoter:GUS (**D**). *PtrCslD5* promoter:GUS (**E**). *PtrCslD8* promoter:GUS (**F**). *PtrCslD9* promoter:GUS (**G**). *AtCslD2* promoter:GUS (**H**). Scale bar unit is μm.

We used the β-glucuronidase (GUS) gene driven by these 10 *CSLD* gene promoters to provide more detailed information about their gene expression patterns. About 2.3–3.4 kb of the promoter regions upstream start codon were amplified and the promoter:GUS was transformed into Arabidopsis. GUS signals were stained in roots, leaves, xylem and flowers (petal and style) in both *pPtrCslD1:GUS* and *pPtrCslD2:GUS* transgenic Arabidopsis (Fig. 3A,B). Strong signals were observed in root tips and leaf vascular tissues in both transgenics, but GUS signals were observed only in root hairs of *pPtrCslD2:GUS* transgenics (Fig. 3A,B). *PtrCslD4 promoter*-driven GUS signals were detected in various tissues, including root hairs, vascular tissues of leaves and petals, vascular bundles of stems, and pollen grains (Fig. 3D). Compared to the *PtrCslD4* promoter, the *PtrCslD3* promoter only gave weak GUS signals in phloem and vascular tissue of leaves (Fig. 3C). GUS staining showed both *PtrCslD5* and *PtrCslD8* promoters were activated in root hairs (Fig. 3E,F), and *PtrCslD5* promoter-driven GUS expression was also detected in pollen sac (Fig. 3E). The observed GUS signals in Arabidopsis root hairs were consistent with the high transcript abundance determined by qRT-PCR (Fig. 2H). In *pPtrCslD9:GUS* transgenic Arabidopsis, GUS signals were detected only in pollen grains (Fig. 3G). We did not observe any GUS signals in the transgenic Arabidopsis of *pPtrCslD6:GUS* and *pPtrCslD7:GUS*.

**Figure 4:**
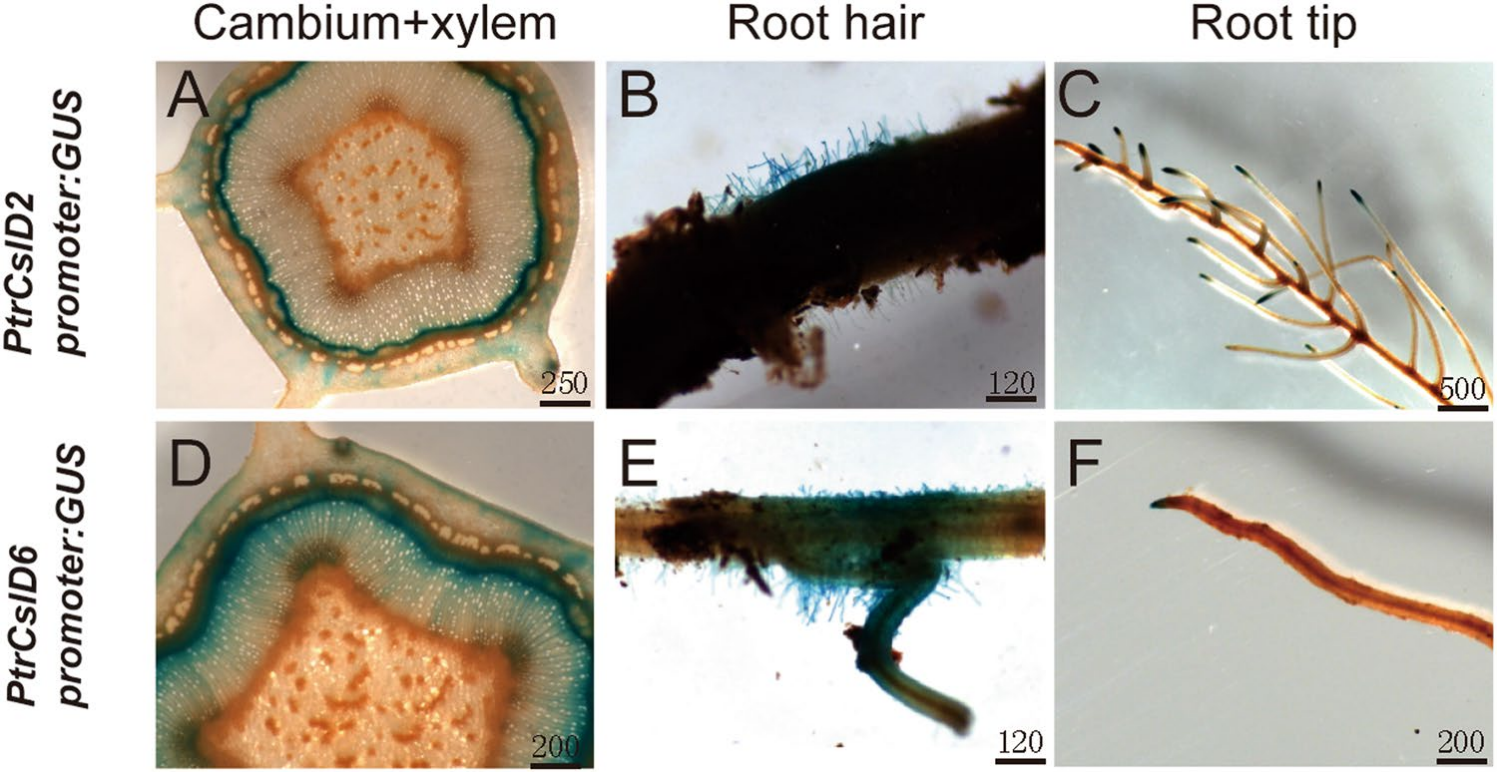
GUS staining of promoter:GUS in *P. trichocarpa*. GUS signals were detected in cambium and xylem (**A**), root hair (**B**) and root tips (**C**) for *PtrCslD2* promoter:GUS. GUS signals were also detected in cambium and xylem (**D**), root hair (**E**) and root tips (**F**) for *PtrCslD6* promoter:GUS. Scale bar unit is μm.

*pPtrCslD2:GUS* in Arabidopsis had GUS signals in root hairs, but *pPtrCslD2:GUS* did not, indicating *PtrCslD2* might express in root hairs of *P. trichocarpa*. To confirm the expression of *PtrCslD2* in root hairs of *P. trichocarpa*, we transformed *pPtrCslD2:GUS* into *P. trichocarpa*. GUS staining in *P. trichocarpa* showed strong signals in developing xylem, root hairs, and root tips (Fig. 4A–C), consistent with the GUS staining in Arabidopsis. Both *PtrCslD6* and *PtrCslD7* promoters did not give GUS signals in Arabidopsis. We selected the *PtrCslD6* promoter to test its ability in *P. trichocarpa*. GUS was stained in xylem, root hairs and root tips in *pPtrCslD6:GUS* transgenic poplar (Fig. 4D–F).

### Complementation to the *atcsld3* mutant

**Figure 5:**
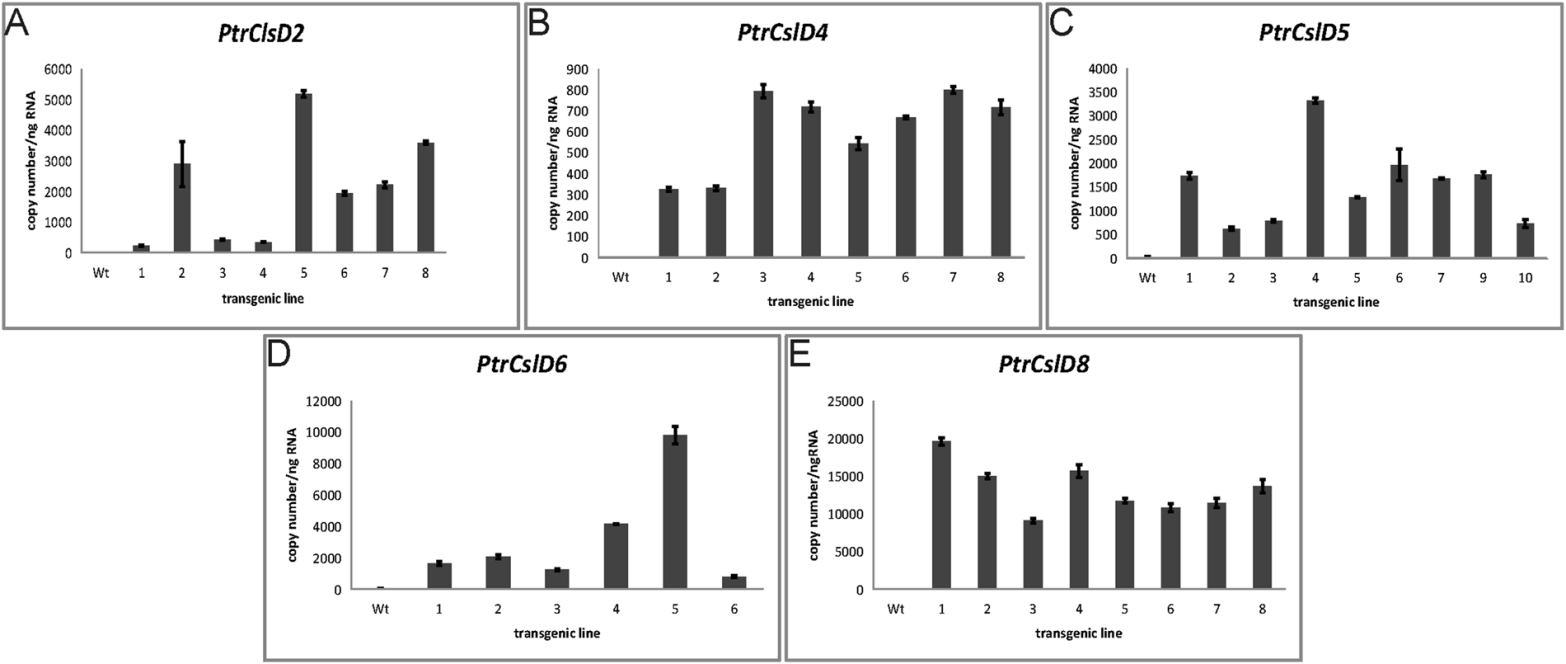
Transgene expression level in *atcsld3* complemented roots. The *atcsld3* mutant was complemented with *PtrCslD2* (**A**), *PtrCslD4* (**B**), *PtrCslD5* (**C**), *PtrCslD6* (**D**), and *PtrCslD8* (**E**). 6–10 transgenic lines were examined for absolute transcript abundance of the transgene in roots.

**Figure 6:**
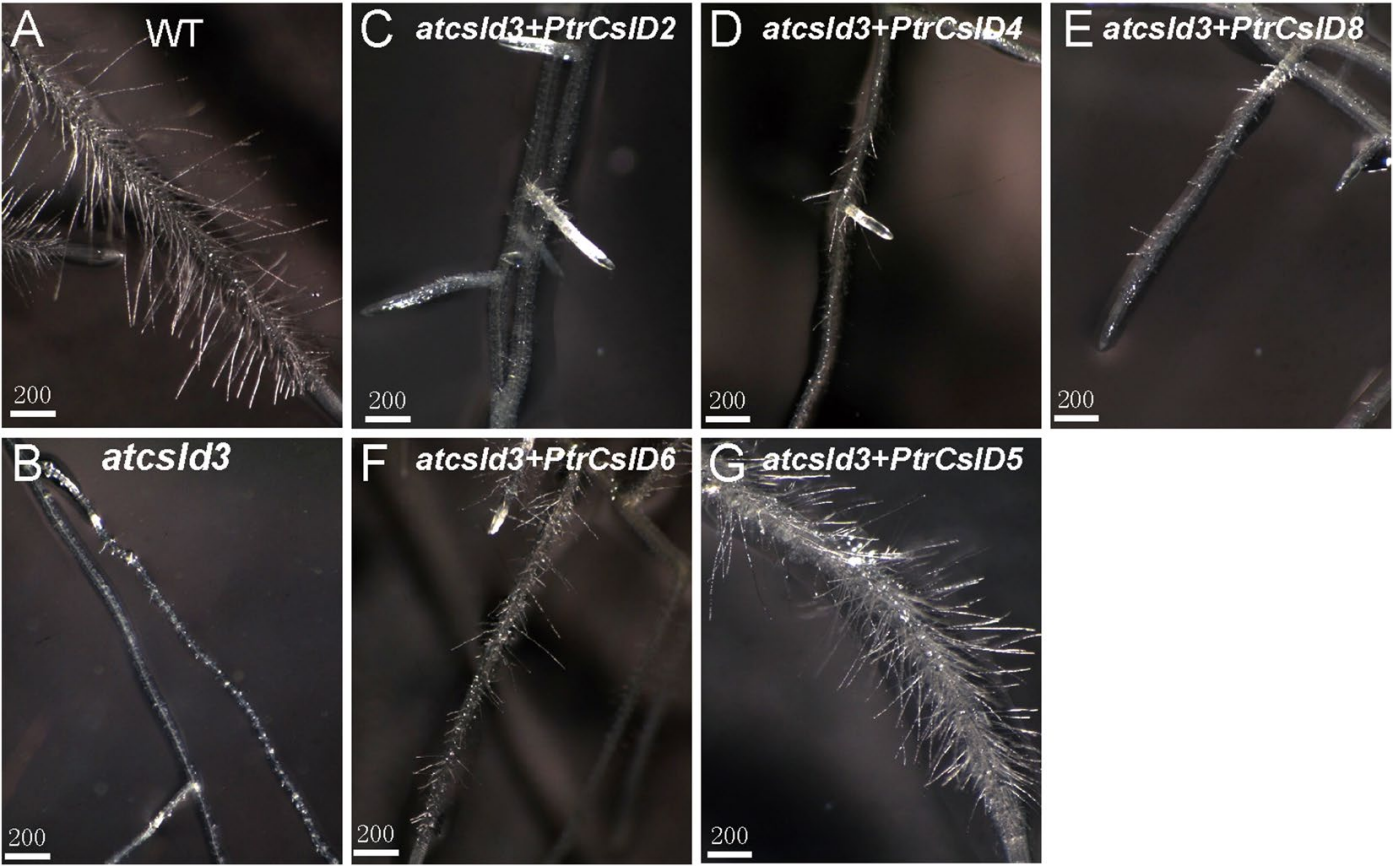
Complementation to the *atcsld3* mutant. Comparison of root hair phenotype among wildtype (**A**), *atcsld3* (**B**), *atcsld3* complemented by PtrCslD2 (**C**), *atcsld3* complemented by PtrCslD4 (**D**), *atcsld3* complemented by PtrCslD8 (**E**), *atcsld3* complemented by PtrCslD6 (**F**), and *atcsld3* complemented by PtrCslD5 (**G**). Scale bar unit is μm.

In the above promoter:GUS experiments, we observed GUS signals in root hairs in several transgenics. The promoters of *PtrCslD2*, *PtrCslD4*, *PtrCslD5*, and *PtrCslD8* could drive GUS expression in Arabidopsis (Fig. 3). Transformation of *PtrCslD2* and *PtrCslD6* promoter-driven GUS in *P. trichocarpa* gave GUS signals in root hairs. To identify the functions of these five *CSLD* genes in root hairs, we overexpressed these genes in the root hair mutant *atcsld3*. Absolute transcript abundance of transgenes in the complementation Arabidopsis was determined by qRT-PCR to confirm their expression (Fig. 5). Numerous root hairs were observed in wildtype Arabidopsis (Fig. 6A). In the *atcsld3* mutant, root hairs were hardly seen (Fig. 6B). Complementation of *atcsld3* with *PtrCslD2*, *PtrCslD4* and *PtrCslD8* gave only a few short root hairs (Fig. 6C–E). Compared with *PtrCslD2*, *PtrCslD4* and *PtrCslD8*, overexpression of *PtrCslD6* in *atcsld3* produced more and longer root hairs, but the root hair number was much fewer and root hairs were shorter than in wildtype (Fig. 6F). *PtrCslD5* complemented mutants appeared with bunches of root hairs (Fig. 6G), and the root hair length was the same as in the wildtype, indicating that *PtrCslD5* is the functional ortholog of *AtCslD3*.

## Discussion

Root hairs, tubular appendages of trichoblast cells of rhizodermis are integral for plant growth, soil anchorage, water and mineral adsorption, symbiotic interface for mychorhizae and nitrogen-fixing bacteria. *Arabidopsis thaliana* root epidermis cells are being used as a model to study cell growth and function^1,2,14,48–50^. Rapid axillary mitotic division of root hairs requires proper cellulose deposition^1,14^. *Cellulose synthase A* (*CesA*) family members are responsible for cellulose biosynthesis, while *cellulose synthesis like D* (*CSLD*) family members are involved in cellulose deposition in both primary and secondary cell walls^5^. Disruption of cellulose deposition would affect root hair formation. In Arabidopsis, several *CSLD* members, including *AtCslD2*, *AtCslD3* and *AtCslD5*, have been characterized for their functions in root hair formation^33^. The root hairless phenotype was observed in the *atcsld3* mutant^31^, and mutant characterization showed that *AtCslD3* is functioning in the initiation of root hair formation^33^. *AtCslD2* is functioning at a later stage of root hair development, and the *atcsld2* mutant had abnormal root hairs, with many rupturing late in development^43^. *AtCslD5* has functions redundant with *AtCslD2* and *AtCslD3*^33^. In our studies on *CSLD* members in *P. trichocarpa*, only *PtrCslD5* could restore the root hairless phenotype of *atcsld3*, indicating that *PtrCslD5* is the functional ortholog of *AtCslD3*. However, *PtrCslD6* shared 96% amino acid sequence identity with *PtrCslD5* but had very little complementation with *atcsld3*. In another study, both *PdCslD5* and *PdCslD6* from *P. deltoids* could rescue the root hairless phenotype in the *atcsld3* mutation^34^. The difference on the complementation to *atcsld3* between *PtrCslD6* and the other three *CSLD* genes (*PtrCslD5*, *PdCslD5* and *PdCslD6*) indicates that some key amino acids may be changed in *PtrCslD6*, reducing its function in root hair formation. PtrCslD6 and PdCslD6 share a 99% amino acid identity, with two amino acid difference (valine vesus isoleucine, and glycine versus serine). It will be interesting to further investigate the roles of these two amino acids in root hair formation. Based on the GUS staining in promoter:GUS transgenic Arabidopsis and poplar, *PtrCslD2*, *PtrCslD4, PtrCslD6, and PtrCslD8* may also function in root hair formation. It is interesting that the GUS expression driven by *PtrCslD5* and *PtrCslD8* promoters were at the same places, root hair and epidermis of root hair zone, but *PtrCslD8* did not restore the phenotype of *atcsld3*. GUS staining shows that *PtrCslD2* promoter activity is induced at an early stage of root hair development, while *PtrCslD4* promoter activity is induced at a later stage of root hair development, suggesting *PtrCslD2* and *PtrCslD4* may function at different stages of root hair development. The functions of *PtrCslD2*, *PtrCslD4*, *PtrCslD6*, and *PtrCslD8* genes in root hair formation and whether they are functioning cooperatively with *PtrCslD5* need further studies, such as complementation to *atcsld2* and *atcsld5* mutants or knockout poplar mutant generation.

In Arabidopsis and other species, *CSLD* members function not only in root hair formation but also in other tissues, such as vascular tissues and pollen^35^. Strong GUS staining of *PtrCslD1* and *PtrCslD2* promoters was observed in vascular tissue of leaves and vascular bundles of stems (Fig. 3A,B), and comparatively light GUS signals in vascular tissue were detected for *PtrCslD3* and *PtrCslD4* promoters (Fig. 3C,D). Phylogenetic tree analysis shows *PtrCslD1* and *PtrCslD2* are in the clade with *AtCslD5* and *OsCslD4*, and *PtrCslD3* and *PtrCslD4* are in the same clade with *AtCslD6*. Triple mutant *csld2/csld3/csld5* had asymmetric loops and discontinuous vascular elements, showing that *AtCslD5*, a gene important for root hair formation, is also functioning in vascular tissues^33^. *OsCslD4* is expressed in the apex of many organs with rapid growth, and its mutation had many effects, such as inhibited plant growth, thin culms, small grains etc^37^. In clade IV, *AtCslD1* and *AtCslD4* are closely related to gene pair *PtrCslD9/10* and gene pair *PtrCslD7/8*. Both *AtCslD1* and *AtCslD4* are important for pollen tube growth^35^. Combining the gene expression patterns and functional characterizations of these 10 *PtrCSLD* genes and other *CSLD* genes in other species, we found that some *CSLD* genes are functioning in multiple tissues, such as root hairs, pollen tubes and vascular tissues. For example, the qRT-PCR and promoter:GUS staining experiment (Figs 2 and 4) showed that *PtrCslD2* was expressed in various tissues, including root hairs, root tips, and xylem. The expression is in accordance with *AtCslD5* expression in Arabidopsis^15^. The expression of *PtrCslD6* was observed in root hairs, root tip, and xylem in *P. trichocarpa*, indicating its roles in the root hairs, root tip and xylem. The expression pattern and predicted function of *PtrCslD6* are in accordance with the functions of *AtCslD2*, *AtCslD3* and *AtCslD5* in root hairs, xylem and tip growth^33^. Although *PtrCslD2* and *PtrCslD6* promoters also drove GUS signals in the *P. trichocarpa* cambium that is lacking in Arabidopsis stems, we assume the expression in cambium is in accordance with the expression of *AtCslD2, AtCslD3* and *AtCslD5* in tip tissue^51^ (Fig. 3H). These results indicate that the *CSLD* genes share a certain level of conservation between Arabidopsis and poplar, and the *CSLD* genes in *P. trichocarpa* may play roles in the same tissues of root hair, vascular tissue and pollen tube, as in Arabidopsis.

We also observed difference between Arabidopsis and poplar related to *CSLD* gene functions and regulation. The activities of the promoters of *PtrCslD1* to *PtrCslD9* were studied in Arabidopsis through promoter:GUS experiments. Surprisingly, no GUS staining was observed for *PtrCslD6* and *PtrCslD7* promoters. However, the *PtrCslD6* promoter was active in *P. trichocarpa*, with staining in root hairs, root tips, cambium, and xylem (Fig. 4). This difference indicates that the upstream regulator(s) of *PtrCslD6* between Arabidopsis and poplar may be different. *PtrCslD1* and *PtrCslD2* transcripts were detected at a very low level in leaves in *P. trichocarpa* (Fig. 2A,B), but both promoters gave strong signals in Arabidopsis leaves (Fig. 3A,B), indicating that the promoters are activated differently between Arabidopsis and poplar. Besides, the expression level of *PtrCslD1* in young roots was higher than that in mature roots, but *PtrCslD2* displayed an opposite expression pattern in young roots and mature roots. The occurrence of the different expression patterns between *PtrCslD1* and *PtrCslD2* in roots may be formed after chromosome duplication.

## Methods

### Plant Materials and Growth

*Populus trichocarpa* (Nisqually-1) were obtained from tissue culture and grown on Murashige and Skoog (MS) medium on 16 h/8 h light and dark under aseptic conditions at 25–28 °C as described previously^52^. Seeds of the *Arabidopsis thaliana atcsld3* mutant line (AT3G03050) were obtained from Nottingham Arabidopsis Stock Centre (NASC, Nottingham, UK). The seeds were surface-sterilized with sterilizing solution (0.1% Trition and 20% NaClO) for 12 minutes, washed with sterilized distilled water and sown on the solid medium containing MS salts for three days before the seeds were put into an illumination incubator at 22 °C with fluorescent white light at 16/8 h light and dark cycles.

### Bioinformatics analysis

The *CSLD* family genes of *A. thaliana* were downloaded from the Arabidopsis Tair database (https://www.arabidopsis.org/) and blasted in *P. trichocarpa* genome via BLASTn search tool with E-value ≤ 1.0. The homologous gene sequences were downloaded from the Phytozome 10.1 plant genomics portal (https://phytozome.jgi.doe.gov/pz/portal.html). We also downloaded *CSLD* family genes already characterized in different species from the NCBI (https://www.ncbi.nlm.nih.gov/gene) database. The unrooted phylogenetic tree for multiple alignment analysis of protein sequences predicted from cDNA sequences of *A. thaliana*, *G. hirsutum*, *G. arboreum*, *G. raimondii*, *O. sativa*, *Z. mays*, and *P. trichocarpa CSLD* genes was constructed with the MEGA 7.0 tool using the Neighbor-Joining (NJ) method through 2000 bootstrap replicates^40^. Each protein encoded by the *P. trichocarpa CLSD* gene family was assigned a specific name according to Van Erp and Walton^39^.

### Quantitative reverse transcription and PCR (qRT-PCR)

For the *PtrCslD* gene expression pattern analysis, leaves, shoot apices, young stems of 1–3 internodes, xylem, phloem, young roots and mature roots were collected from six-month-old trees and put in liquid nitrogen immediately. Total RNA was extracted using the CTAB method^53^. For the qRT-PCR analysis of *PtrCslD* genes in the *atcsld3* mutant, the total RNA was extracted from the roots using an RNeasy Plant Mini Kit (Qiagen). The reverse transcription of RNA to cDNA and quantitative polymerase chain reaction (PCR) were carried out as described previously^52^. The primers used in the qRT-PCR are listed in Supplemental Table S1.

### Promoter-driven *GUS* expression in Arabidopsis and *P. trichocarpa*

The promoter regions of 2.4–3.4 kb upstream start codon were amplified for *PtrCSLD* genes using specific primers (Supplemental Table S1). The sizes of amplified fragments were 3.44 kb (*PtrCslD1*), 2.34 kb (*PtrCslD2*), 2.73 kb (*PtrCslD3*), 2.73 kb (*PtrCslD4*), 2.5 kb (*PtrCslD5*), 2.7 kb (*PtrCslD6*), 2.75 kb (*PtrCslD7*), 2.78 kb (*PtrCslD8*), 2.8 kb (*PtrCslD9*), and 2.67 kb (*PtrCslD10*). The promoters of *PtrCslD1* to *PtrCslD9* were successfully amplified. The amplified fragments were cloned into *pCR2.1* for sequencing. Further, the promoter fragments were excised from pCR2.1 vectors and inserted into *pBI121* by replacing the 35 S promoter, generating *pPtrCslD1:GUS*, *pPtrCslD2:GUS*, *pPtrCslD3:GUS*, *pPtrCslD4:GUS*, *pPtrCslD5:GUS*, *pPtrCslD6:GUS*, *pPtrCslD7:GUS*, *pPtrCslD8:GUS*, and *pPtrCslD9:GUS*. All constructs were introduced into the *Agrobacterium tumefaciens* strain GV3101. Transformation in Arabidopsis followed the floral dip method^54^. T1 transgenic plants were screened on MS plates with 30 mg/L kanamycin and transferred to MS plates without kanamycin. *Agrobacterium*-mediated transformation in *P. trichocarpa* was conducted using 5–8 internode stems as explants following the previous publication^55^. After being verified by PCR using DNA as templates, the transgenic plants were moved into pots and maintained in a greenhouse. GUS staining and observation were conducted as described previously^52^.

### Mutant complementation

The total RNA isolated from the xylem for qRT-PCR analysis was reverse-transcribed to cDNA using an Omniscript RT kit (Qiagen). Using the cDNA as templates, the full-length cDNAs of *PtrCslD2*, *PtrCslD4*, *PtrCslD5*, *PtrCslD6* and *PtrCslD8* were amplified with designed primers (Supplemental Table S1). The PCR fragments were inserted into *pBI121* to replace the *GUS* gene, generating *35 S:PtrCslD2*, *35 S:PtrCslD4*, *35 S:PtrCslD5*, *35 S:PtrCslD6*, and *35 S:PtrCslD8*. After transformation in the *atcsld3* mutant by floral dip method, 6–10 lines were confirmed for transgene expression in roots by qRT-PCR as described above. The root hairs in the wildtype, *atcsld3* mutant, and complementation plants were photographed under a Zeiss (Stemi DV4) microscope.

## Electronic supplementary material

Supplementary Information
