# Supplementary material for: Characterization of Cellulose synthase-like D (CSLD) family revealed the involvement of PtrCslD5 in root hair formation in Populus trichocarpa
Source: Sci Rep. 2019 Feb 5;9:1452. doi: 10.1038/s41598-018-36529-3 (PMC6363781; doi:10.1038/s41598-018-36529-3)
Supplement: Supplementary file 1 — Supplementary Information [file 41598_2018_36529_MOESM1_ESM.docx]

**Supplementary Information**

**Characterization of *Cellulose synthase-like D* (*CSLD*) family revealed the involvement of *PtrCslD5* in root hair formation in *Populus trichocarpa***

Xiaopeng Peng^1#^, Hongying Pang^1#^, Manzar Abbas^1,3#^, Xiaojing Yan^1^, Xinren Dai^1^, Yun Li^3^, Quanzi Li^1,2,^*

^1^State Key Laboratory of Chinese Academy of Forestry, Beijing 100091, China

^2^Research Institute of Forestry, Chinese Academy of Forestry, 100091, Beijing, China

^3^National Engineering Laboratory for Tree Breeding, College of Biological Sciences and Technology, Beijing Forestry University, Beijing 100083, China

^#^These authors contributed equally to this work.

* Correspondence: Quanzi Li ([liqz@caf.ac.cn](mailto:liqz@caf.ac.cn))

**Table S1** Primers used in this study

| **Primer Name** | **Primer sequences (5'-3')** | **Purpose/Note** |
| --- | --- | --- |
| PtrCslD2-F | GGATCCGATACAATACAAGCCTCAGCATTCG | Full transcript cloning  (Restriction sites are underlined) |
| PtrCslD2-R | GTCGACACATCAAGGGATCTGAAACTTCATG |  |
| PtrCslD4-F | TCTAGAGCTCATATAACCACTTTGATTTCAGG |  |
| PtrCslD4-R | GAGCTCACCTAGAGATAGCAGAAACACGA |  |
| PtrCslD5-F | TCTAGATTTGAAGAGACGTGTCCCTTAC |  |
| PtrCslD5-R | GAGCTCAGACCTATCAGGGAAACTGGAATG |  |
| PtrCslD6-F | CTCTAGAATCATCTCAGTGCAACTAAAGG |  |
| PtrCslD6-R | TGAGCTCTCAAGGAAACTGGAAAGAGCCACCGAT |  |
| PtrCslD8-F | CTCGAGGTGCGTTTTTATTGTGTCATCGA |  |
| PtrCslD8-R | GAGCTCGATTCTTGCATCTGGTGTTCACTC |  |
| PtrCslD9-F | GGATCCACACCATTGTCACAACAAAATCAAT |  |
| PtrCslD9-R | GAGCTCTCGAACTGACATTACACTTGAAG |  |
| PtrCslD9-F | GGATCCATTTCTATGTTGCAGCATTGGTT |  |
| PtrCslD9-R | GAGCTCCCCTCTCCCCCTCAGTCTCGA |  |
| CslD1-rF | TGCACAACAGAGGATGGAGGTCTG | qRT-PCR |
| CslD1-rR | GTTGCCCATCGAAGGACTTGGTGA |  |
| CslD2-rF | CTTGTGAGTTATGGTTTGGTGTGTCA |  |
| CslD2-rR | CCTGGAAGGTCTGATCGGCCTTTT |  |
| CslD3-rF | GCATTTCAAGGTCTTCCCCTCGCA |  |
| CslD3-rR | GCAAGAGATGACATTCACTGCCTCA |  |
| CslD4-rF | GCATTTCAAGGTCTGCCCCTCGCT |  |
| CslD4-rR | GCAAGAGATGACATTCACTGCCTCT |  |
| CslD5-rF | ATGGCCTCAAGATCATTCAAAGGC |  |
| CslD5-rR | CCAAATGTCACGGTCTGAGGTAGA |  |
| CslD6-rF | ATGGCCTCAAAATCATTCAAGGCT |  |
| CslD6-rR | CCAAATGTCACCGACGGAGGCAGG |  |
| CslD7-rF | CGAACTATACGGTGCAGATTCCCTC |  |
| CslD7-rR | TGAGATGCGCACGCGTAACGC |  |
| CslD8-rF | TGTCTCATCCTCAGATGGCTGGAT |  |
| CslD8-rR | TTTGAGCCTGCATTCACAAGGTATGAT |  |
| CslD9-rF | CAGGATACGGAGCTGAGTATGTCC |  |
| CslD9-rR | CCTGTAAGGGCTGAGAATAGCTGCA |  |
| CslD10-rF | CAGGATTCAGAGCTGAGCAAGTCG |  |
| CslD10-rR | TCCTGTAAGGACTGAGAATAGCTGCT |  |
|  |  |  |
| CslD1P-F | GAAGCTTACATAACCAATCTCACCATGCGC | Promoter amplification  (restriction sites are underline) |
| CslD1P-R | TGGATCCAGAACCCAGATGATGGTTGCGGT |  |
| CslD2P-F | CGATATCGATCCATAGTAGTAGTTGATCATCAAAC |  |
| CslD2P-R | AGGATCCTCTCTCACGAGTGGCACATTCG |  |
| CslD3P-F | CAAGCTTTAGGCCCTTGTTAATCCTGTA |  |
| CslD3P-R | TGGATCCTATGAAGAACACAGAGTCTGGG |  |
| CslD4P-F | ACCCGGGCGGATTTAGTTGAACCACAAACG |  |
| CslD4P-R | TGGATCCAGCCCAAAATCAATCCTCTTCTTT |  |
| CslD5P-F | CTCGAGCCTGGCAGTACCTGCTAGACCCAC |  |
| CslD5P-R | GTCGACGGACACGTCTCTTCAAATCCTGCA |  |
| CslD6P-F | CGATATCACTCGCAATCAACCAACCTCATCTG |  |
| CslD6P-R | CGGATCCTCTGCTTAGGGGCCAAGAAACT |  |
| CslD7P-F | GAAGCTTCGGTGACTCGAACTTGTGGGTC |  |
| CslD7P-R | CGGATCCACGATACAACGAACACACCATAACA |  |
| CslD8P-F | GAAGCTTGATGCGCGAGGAGTATCTTCATC |  |
| CslD8P-R | TGGATCCAACATTTGCGCCTCATCATCATCA |  |
| CslD9P-F | CGATATCCCATCACCGACCGAAATTACATCA |  |
| CslD9P-R | CGGATCCGATTTTGTTGTGACAATGGTGTTTTC |  |
| CslD10P-F1 | TCCCGGGTTCCCAGCCAAATGATCCATGTC |  |
| CslD10P-R1 | CGGATCCAGGTTTTGTTGAGGTTGCCATGAC |  |
| CslD10P-F2 | TCCCGGGATGATGGCAGCGTGGTGGCGTAT |  |
| CslD10P-R2 | CGGATCCAGCGGTTGATGCTTGTGATGAC |  |
| AtCslD1P-F | CTCGAGAGATATGGTGGTGAAAAGGCTGC |  |
| AtCslD1P-R | TCTAGAATTATCTTGTTTTGGGTCGTTTGG |  |
| AtCslD2P-F | AAGCTTCTGCAGGGATCTGCCAAGTTAG |  |
| AtCslD2P-R | GGATCCTAACTTGGCAGATCCCTGCAGA |  |
| AtCslD4P-F | AAGCTTGTAGCAGGTCCTGACTAGATGG |  |
| AtCslD4P-R | GGATCCAAACAAAGGAGCTTGGTTTCTGC |  |
| AtCslD6P-F | CTCGAGAGAAGTTCATGCGAACGGCAC |  |
| AtCslD6P-R | GGATCCGTCGCCGGAATCTGGAGAAA |  |
